# Tannate of Quinine

**Published:** 1850-05

**Authors:** R. H. Thomas


### ARTICLE III.

*Tannate of Quinine*.—By R. H. Thomas, M. D.

[The following on a combination of tannic acid with quinine, we extract from the Amer. Med. Journal for April.]

I have prescribed it in a number of instances, and found that whilst it was equally effectual, it was far more palatable than any other combination of quinine I was acquainted with. On referring to the *American Journal of the Medical Sciences*, Vol. XIX. p. 219 (1836), it will be found that Dr. Ronander, Secretary of the Swedish Medical Association, recommended, in 1834, the tannate of quinine and cinchonin as the most active ingredients of the Peruvian bark; he asserts that he has cured by their means, several cases of obstinate ague, which had resisted the use of sulphate of quinine, and other powerful remedies, &c. &c. Nothing is said in the extract, from the original paper in *Hecker's Annals*, December, 1834, of the taste of the tannate of quinine. Compared with the sulphate, it is almost tasteless.

The following is the extemporaneous prescription I am in the habit of ordering for a child two years old: ℞. Quiniæ sulph. gr. x; acid. tannici gr. ij; aqua ℥vj; syrup aurant. ℥ij. M. A teaspoonful every hour or two.

